# Equation for Tooth Size Prediction from Mixed Dentition Analysis for Taiwanese Population: A Pilot Study

**DOI:** 10.3390/ijerph18126356

**Published:** 2021-06-11

**Authors:** See Yen Chong, Lwin Moe Aung, Yu-Hwa Pan, Wei-Jen Chang, Chi-Yang Tsai

**Affiliations:** 1 School of Dentistry, College of Oral Medicine, Taipei Medical University, Taipei 110, Taiwan; m204104002@tmu.edu.tw (S.Y.C.); drlwinmoeaung@gmail.com (L.M.A.); shalom.dc@msa.hinet.net (Y.-H.P.); 2 Department of Dentistry, Chang Gung Memorial Hospital, Taipei 110, Taiwan; 3 Graduate Institute of Dental & Craniofacial Science, Chang Gung University, Taoyuan 333, Taiwan; 4 School of Dentistry, College of Medicine, China Medical University, Taichung 4060, Taiwan; 5 Dental Department, Shuang-Ho Hospital, Taipei Medical University, New Taipei City 235, Taiwan; 6 Dental Department, Taipei Medical University Hospital, Taipei 110, Taiwan

**Keywords:** mixed dentition analysis, Taiwanese, tooth size prediction, Tanaka–Johnston, Moyers’ probability tables, regression equation

## Abstract

Background: In mixed dentition analysis, estimation of the mesiodistal width of unerupted permanent canines and premolars is essential for successful diagnosis and treatment planning. The present study aimed to develop a simple linear equation to predict permanent tooth sizes from mixed dentition analysis for Taiwanese people. Methods: The sample comprised 200 dental casts, derived from Taiwanese patients (100 males and 100 females; age, 12–35 years). Mesial distal tooth widths were measured in dental casts with a digital caliper. A student’s *t*-test was conducted to detect tooth size correlations with gender-specific differences, as well as intra-arch counterparts. Standard linear regression was conducted to develop a simple equation representing predictions of canine-premolar relationships. Results: All teeth were not significantly different between the left and right sides, regardless of gender and upper or lower arches. In terms of types of teeth, males had larger tooth dimensions in both arches than females. New regression equations for estimating the dimensions of the unerupted canines and premolars in the Taiwanese population were developed. Conclusions: Using a sample of Taiwanese people, new models derived for females and males separately were developed, which should provide highly accurate predictions for unerupted canines and premolars in the Taiwanese population.

## 1. Introduction

In Taiwan, the demands for more quality orthodontic treatments continue to increase. The continuous decline in the age of orthodontic patients challenges the ascending significance of mixed dentition space analysis. Recent reviews revealed increasing enthusiasm toward the beginning of orthodontic therapy during the mixed dentition period [1]. The self-awareness of dental irregularities has increased in the current population and early orthodontic treatment has been a trend [2]. Space analysis is common when considering adequate early treatment choices. Additionally, space evaluation and tooth size predictions for mixed dentition can help clinicians justify tooth extractions and anchorage decisions in orthodontic treatment plans [3].

Space analysis is the evaluation of spacing or crowding within the dental arches, which is achieved by comparing the amount of space available with the amount of space required to align the teeth properly. Space analysis aims to compare the arch length with tooth material. Methods used for space analysis include the Nance analysis, Moyers analysis, Tanaka–Johnston analysis, Staley–Kerber analysis, Merrifield analysis, and Bolton analysis [4].

Two of the most commonly used methods of mixed dentition analysis are the Moyers probability table and the Tanaka–Johnston method of prediction [5]. Moyers’ mixed dentition analysis uses measurements taken from the four mandibular incisors to find the estimated size of maxillary and mandibular canines and premolars from probability tables that were calculated for girls and boys. The mandibular incisors were used because they provided greater accuracy than did maxillary lateral incisors, which vary more in size [6].

The Tanaka–Johnston analysis is a variation of the Moyers method [7], except that they introduced simple, easily remembered regression equations to widen its clinical application. In this technique, the total width of the four mandibular permanent incisors is measured and then divided by 2. The result plus 10.5 mm gives the estimated width of the mandibular permanent canine and premolars and the result plus 11.0 mm gives the estimated width of the maxillary canines and premolars. According to Dean et al. [8], the estimated width of unerupted canines and premolars measured with the Tanaka–Johnston method corresponds to the 75% level of probability in the Moyers prediction table. The Tanaka–Johnston analysis thus provides significant clinical acceptability with a minimal amount of time and effort.

However, the original Tanaka–Johnston analysis was conducted on a population of North European descent, including a sample of 506 North American orthodontic patients. It is reasonable to question their use in other populations. Thus, this study aimed to determine the mesiodistal widths of canines and premolars and to check the applicability of the Tanaka–Johnston method in a Taiwanese population.

There have been several studies [5,9,10,11] of mixed dentition space analysis in other population groups that disagreed with the use of the Moyers and Tanaka–Johnston methods. Moreover, there are many questions about applying these methods, which are based on pooled male and female data, rather than considering the genders separately. The literature shows that tooth size varies between males and females with males having larger teeth than females [12,13,14,15,16,17,18,19].

The Tanaka–Johnston prediction equation overestimated the sum of mesiodistal widths of permanent canines and premolars in populations from Saudi Arabia [9,14,20], Turkey [13], Carolina [21], and Bangalore [22]. Additionally, the literature indicates that there is a limitation or low accuracy in the application of the two most commonly used prediction methods in Jordan [12], Thailand [15], India [17,18,23], Syria [24], Brazil [25], and Iran [26].

For the Chinese population, Yuen et al. suggested applying the prediction equation or probability tables developed in their study to improve accuracy in the mixed dentition analysis for southern Chinese [5]. According to Ling et al., gender dimorphism in the mesiodistal dimension was evident between southern Chinese males and females [27]. Sherpa et al. analyzed the applicability of the Tanaka–Johnston and Moyers methods in Northeast Han Chinese and found that the Tanaka–Johnston equations were not precise, except for the upper arch in males. However, Moyers’ method, which was in the 85th percentile in the upper arch and 75th percentile in the lower arch, predicted the sum precisely in males. For females, the Moyers’ 75th percentile predicted the sum precisely for the upper arch, but none of the Moyers’ percentiles provided accurate predictions in the lower arch.

Given that there has been no study on mixed dentition space analysis in a Taiwan population, the objectives of this study were to develop a new regression equation using the formula Y = a + b (X) and to determine gender dimorphism with respect to the Tanaka–Johnston mixed dentition analysis in a Taiwanese population.

## 2. Materials and Methods

### 2.1. Subjects

The subjects in this cross-sectional study were selected from among 1025 patients of the Orthodontic Branch, Dental Department of Taipei Medical University Hospital.

### 2.2. Sample Size Calculation

Using the sample size calculator developed by Creative Research Systems survey software (Copyright © 2012 Creative Research Systems, Petaluma, CA, USA), we found that 385 or more subjects were needed to have a confidence level of 95% and that the real value was within ±5% of the measured value. However, after we examined the patients in the age range of 12–35 years, only 200 subjects (100 males and 100 females) met our study criteria. The margin of error of this sample size was 6.79%, which means that there was a 95% chance that the real value was within ±6.79% of the measured value.

### 2.3. Inclusion and Exclusion Criteria

Inclusion criteria were as follows: the person was a Taiwanese descendant, all permanent teeth were present in each arch (fully erupted except for the second and third molars), and there was moderate crowding and spacing of teeth (<10 mm). The exclusion criteria were as follows: subjects with congenital craniofacial anomalies, congenital missing or previous orthodontic treatment, and teeth with proximal caries, proximal restorations, tooth fractures, proximal/occlusal abrasions, and bruxism. The study was conducted according to the guidelines of the Declaration of Helsinki and approved by the Institutional Review Board of Taipei Medical University (TMU-JIRB N202006038).

### 2.4. Experimental Procedure

Two investigators measured the un-soaped plaster study models manually and independently. The mesiodistal widths of all permanent incisors, canines, premolars, and first molars were measured with digital calipers (range, 0–150 mm; accuracy, ±0.01 mm; Mitutoyo Corporation, Tokyo, Japan) and were read to the nearest 0.01 mm. The beaks of the calipers were machine sharpened to a fine taper to improve accessibility to the proximal surfaces of the teeth, especially for the mesiodistal dimensions.

All measurements were made perpendicular to the long axis of the tooth, with the beaks entering the interproximal area from either the buccal or the occlusal side. The preferred method was from the buccal side unless the tooth appeared to be severely rotated. Interexaminer and intraexaminer reliabilities were predetermined at 0.2 mm, as suggested by Bishara et al. [28]. The two measurements obtained by the investigators were compared; if less than a 0.2 mm variation was found, then the values were averaged. If there was more than 0.2 mm variation, the teeth were remeasured and the closest three measurements were averaged. Intraexaminer and interexaminer variabilities were obtained by measuring 20 sets of randomly selected dental casts twice in 2-week intervals [29]. Dahlberg’s formula [30] was used to quantify the measurement error:

$$D=\sqrt{\sum_{i=1}^{N}\frac{d_{i}^{2}}{2N}}$$

where *d_i_* is the difference between the original measurement value and the repeated-measurement value, and *N* is the sample size which was remeasured.

### 2.5. Statistics

Descriptive statistics, including means, standard deviations, and ranges, were calculated for the teeth (from permanent incisors to first molars of both arches). Student’s *t*-tests were used to determine whether there were significant differences between the right and left sides in each arch for the boys and girls, as well as between the genders using an independent samples *t*-test. Correlation coefficients and regression equations were formulated to observe any relationship between the summed widths of the four mandibular incisors and the canines and premolars of each dental arch. Statistical calculations and analyses, including standard errors of the estimation and coefficients of determination, were conducted using Statistical Analysis Software (SAS/SAT version 9.4 for Windows, SAS Inc., Cary, NC, USA).

## 3. Results

**Figure 1:**
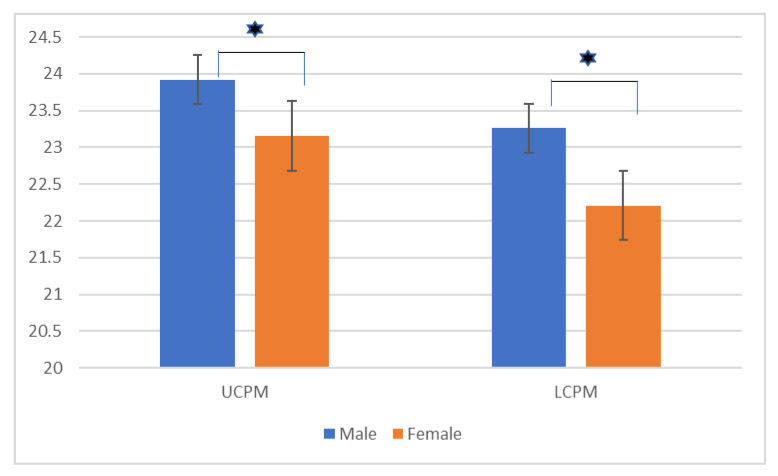
Comparison of tooth sizes between males and females (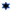 = *p* < 0.05; UCPM = combined mesiodistal width of the upper canines and premolars; LCPM = combined mesiodistal width of the lower canines and premolars).

The mean age of males and females was 19.3 ± 5.6 and 20.1 ± 7.0 years old (Table 1), respectively. After quantifying measurement error with Dahlberg’s method, the error values of the mesiodistal widths ranged from 0.1mm to 0.25mm and were considered clinically acceptable. No statistically significant tooth size difference was found between intra-arch counterparts but such significant differences existed between genders (*t*-test, *p* < 0.05), with males having larger teeth than females (Figure 1, Table 2).

Using a paired sample *t*-test, we found that the Tanaka–Johnston analysis overestimated the combined mesiodistal width of canines and premolars in the upper and lower arches in both genders (*p* < 0.001), except for the female lower arch, which showed no significant difference between the actual tooth size and tooth size predicted by the Tanaka–Johnston analysis (Table 3).

**Figure 2:**
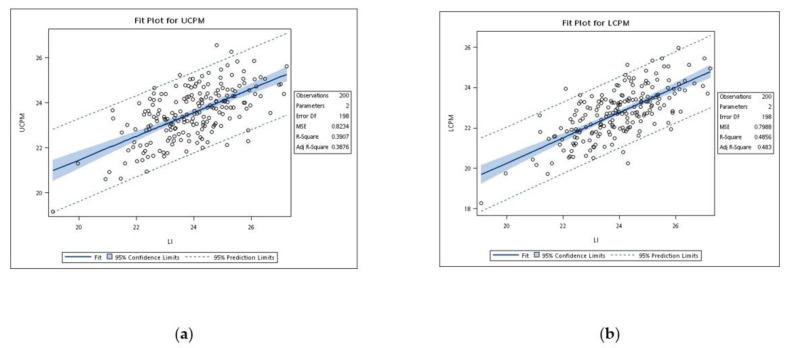
(**a**) Regression of actual tooth sizes of the upper canines and premolars and the sum of mandibular incisors for both genders; (**b**) regression of actual tooth sizes of lower canines and premolars and the sum of mandibular incisors for both genders.

**Figure 3:**
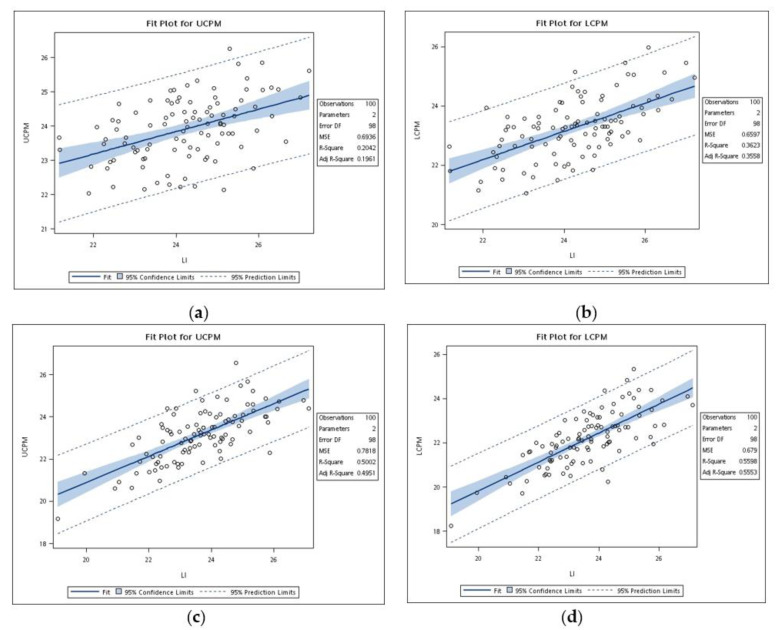
(**a**) Regression of actual tooth sizes of the upper canines and premolars and the sum of mandibular incisors for males; (**b**) regression of actual tooth sizes of lower canines and premolars and the sum of mandibular incisors for males; (**c**) regression of actual tooth sizes of upper canines and premolars and the sum of mandibular incisors for females; (**d**) regression of actual tooth sizes of lower canines and premolars and the sum of mandibular incisors for females.

Because of the tooth size differences between males and females, as well as upper and lower arches, six regression models based on the sum of mandibular incisors and actual tooth sizes of canines and premolars were developed (Figure 2 and Figure 3). All six linear regression equations were statistically significant (*p* < 0.001). Table 4 shows the regression parameters for predictions of the combined mesiodistal width of canines and premolars from the sum of the width of mandibular incisors. The predictive accuracy of a regression equation was indicated by the coefficient of determination (r^2^). In this study, r^2^ values ranged from 0.20 to 0.56. The r^2^ values of females were higher than males, with the figures for females consistently better than those for males. The standard errors of estimation ranged from 0.81 to 0.90 mm. According to these parameters, prediction equations for males or females are described in Table 5.

## 4. Discussion

This study performed an evaluation of a new regression equation for mixed dentition analysis in a Taiwanese population. After we examined the patients in the age range of 12–35 years, two investigators selected 200 subjects (100 males and 100 females) who met our study criteria. However, according to the sample size calculation, 385 or more subjects were needed to have a confidence level of 95% and that the real value was within ±5% of the measured value. Therefore, after this pilot study, increasing the sample size is mandatory for a full-scale research project.

Although the age range of our enrolled subjects was large, all the measurements were performed in permanent dentition and any changes in mesiodistal size of permanent teeth, such as abrasion, attrition, etc., have been excluded in this study.

According to the results of the present study, all teeth were not significantly different between the left and right sides regardless of gender and the upper or lower arches. In terms of types of teeth, males had larger tooth dimensions in both arches. The new regression equations for estimating the dimensions of the unerupted canines and premolars in a Taiwanese population were developed.

The primary factor that causes space anomalies during the development of occlusion is the mesiodistal tooth width, which, together with tooth width discrepancy, may cause malocclusion [31,32,33]. Therefore, it is essential to conduct mixed dentition space analysis before any orthodontic treatment plan is created.

Melgaço et al. [34] mentioned that radiographic methods (e.g., periapical X-ray and 45° oblique radiographs), nonradiographic methods (e.g., prediction tables and regression equations), and combination methods (e.g., Staley and Kreber analysis and method of Hixon and Oldfather) are the three commonly used methods for mixed dentition analysis. Carlos et al. showed that in comparison to actual tooth lengths, conventional panoramic radiographs were relatively inaccurate, overestimating the lengths by 29%, while CBCT panoramic reconstructions underestimated the lengths by 4% [35]. Because the patient’s awareness of radiation exposure and radiographic methods are less practical in nature, we adopted nonradiographic methods in this study. Although digital models are an accurate, efficient, and easy-to-use alternative to plaster models [36], some scholars believe that there are no statistically significant differences between the values measured by manual methods (plaster models) and digital methods (digital models) [37,38]. 

Our study showed that, with respect to intra-arches, the tooth sizes were not significantly different between the right and left sides but varied among both males and females. The findings of the present study, which showed that males have larger teeth than females, were similar to other studies [12,13,14,15,16,17,19,39]. However, a recent study regarding the Hong Kong population found that, for the mesiodistal width of the lower lateral incisor, there was no significant difference between males and females [27]. In this study, the result indicating that the Taiwanese sample had larger tooth dimensions than the Caucasian sample was similar to those of the other studies [5,40].

The regression coefficient, also called the slope coefficient, determines the strength of the relationship between the independent and dependent variables of the regression line. The regression coefficients in this study, which ranged from 0.33 to 0.65, were smaller than those found for Caucasians [6,7,41,42]. By contrast, larger regression coefficients were found in the Hong Kong population during the construction of the regression equation for predictions of unerupted permanent teeth [5]. Since the results from the regression analysis for females were consistently better than those for males, we concluded that the prediction models for females were more precise than for males because of the higher value of the coefficient of determination (r^2^) in the female regression model. However, other studies [43,44], including one study from Hong Kong [5], drew the opposite conclusion.

Our study also revealed that the Tanaka–Johnston analysis significantly underestimated the width of the unerupted canines and premolars, except for mandibular canines and premolars of females. Therefore, there was a need to construct new regression equations for predictions of unerupted canines and premolars in the Taiwanese population.

When comparing the regression parameters among the different populations to inspect the representation of our study, we found a high-to-medium correlation in the female group and a medium-to-lower correlation in the male group (Table 6). Correlation coefficients in our study were similar to those of previous studies, so these regression parameters can be put into good clinical orthodontic use by the construction of prediction equations for a Taiwanese sample.

## 5. Conclusions

Significant differences in tooth sizes for different genders were found in the Taiwanese population. The Tanaka–Johnston analysis overestimated the combined mesiodistal width of the canines and premolars in the upper and lower arches for both genders (*p* < 0.001) in the Taiwanese population, except for female lower arches. For Taiwanese patients, new regression equations were derived in this study for predictions as follow:

Male

Upper: y = 15.99 + 0.33x

Lower: y = 11.75 + 0.47x

Female

Upper y = 8.42 + 0.62x

Lower y = 6.73 + 0.65x

(x = combined mesiodistal width of the lower incisors; y = combined mesiodistal width of canines and premolars)

Using a Taiwanese sample, the new models developed in this study were derived for females and males separately, which should provide highly accurate predictions for unerupted canines and premolars in the Taiwanese population.

## Figures and Tables

**Table 1:** Demographic profile of this study.

Gender	Mean Age (Years Old)	*n*
Males	19.3 ± 5.6	100
Females	20.1 ± 7.0	100

**Table 2:** Comparison of tooth sizes between males and females.

Teeth	Male	Female
13, 23	8.47 ± 0.37	8.14 ± 0.43 *
14, 24	7.97 ± 0.35	7.69 ± 0.48 *
15, 25	7.48 ± 0.39	7.30 ± 0.48 *
31, 41	5.77 ± 0.35	5.60 ± 0.34 *
32, 42	6.35 ± 0.35	6.23 ± 0.40 *
33, 43	7.50 ± 0.38	7.01 ± 0.40 *
34, 44	7.92 ± 0.35	7.61 ± 0.44 *
35, 45	7.84 ± 0.45	7.59 ± 0.51 *

* *p* < 0.05.

**Table 3:** Actual tooth size and tooth size predicted by the Tanaka–Johnston analysis.

Arch	Actual Tooth Size	Tanaka–Johnston Analysis
Upper		
CPM (M + F)	23.54 ± 1.16	22.97 ± 0.69 *
Lower		
CPM (M + F)	22.73 ± 1.24	22.47 ± 0.69 *
Upper		
CPM (M)	23.92 ± 0.92	23.11 ± 0.64 *
Lower		
CPM (M)	23.25 ± 1.01	22.61 ± 0.64 *
Upper		
CPM (F)	23.16 ± 1.24	22.82 ± 0.70 *
Lower		
CPM (F)	22.21 ± 1.23	22.32 ± 0.70

M = male; F = female; CPM = combined mesiodistal width of the canines and premolars; * *p* < 0.01.

**Table 4:** Regression parameters for predictions of the combined mesiodistal width of canines and premolars and the sum of widths of mandibular incisors.

Group	Arch	r	a	b	SEE	r^2^	
Male + Female	Upper	0.63	10.94	0.53	0.90	0.39	*
	Lower	0.70	7.68	0.63	0.90	0.49	*
Male	Upper	0.45	15.99	0.33	0.83	0.20	*
	Lower	0.60	11.75	0.47	0.81	0.36	*
Female	Upper	0.71	8.42	0.62	0.88	0.50	*
	Lower	0.75	6.73	0.65	0.82	0.56	*

* *p* < 0.01.

**Table 5:** Regression equations.

Arch	Group	Equation
Upper	Male	Y = 15.99 + 0.33X
	Female	Y = 8.42 + 0.62X
Lower	Male	Y = 11.75 + 0.47X
	Female	Y = 6.73 + 0.65X

**Table 6:** Comparison of regression parameters among different populations.

Study	Y	r	a	b	SEE	r^2^
Taiwan, 2021	Md-M	0.60	11.75	0.47	0.81	0.36
	Mx-M	0.45	15.99	0.33	0.83	0.20
	Md-F	0.75	6.73	0.65	0.82	0.56
	Mx-F	0.71	8.42	0.62	0.88	0.50
North America, 1974 [7]	Md	0.65	9.18	0.54	0.85	0.42
	Mx	0.63	10.41	0.51	0.86	0.40
Hong Kong, 1998 [5]	Md-M	0.77	8.82	0.58	0.61	0.60
	Mx-M	0.79	7.97	0.66	0.68	0.62
	Md-F	0.69	6.66	0.64	0.82	0.47
	Mx-F	0.65	8.30	0.61	0.81	0.42
Turkey, 2009 [13]	Md-M	0.98	4.51	0.71	0.01	0.96
	Mx-M	0.98	5.32	0.71	0.01	0.96
	Md-F	0.97	4.17	0.73	0.02	0.94
	Mx-F	0.96	3.82	0.78	0.02	0.91
Karachi, 2011 [45]	Md-M	0.54	12.09	0.44	0.84	0.29
	Mx-M	0.71	11.14	0.48	0.58	0.51
	Md-F	0.88	6.65	0.65	0.55	0.77
	Mx-F	0.88	10.22	0.51	0.42	0.77

Md = mandibular canines and premolars; Mx = maxillary canines and premolars; M = male; F = female.

## Data Availability

The data presented in this study are available on request from the corresponding author.
